# Iron and Alum Mass

**Published:** 1877-07-20

**Authors:** 


IRON AND ALUM MASS.—There is sufficient evidence, drawn from actual experiment in the use of this article, to show that it is a very valuable agent, particularly in chronic disorders of the liver, stomach, and uterus. The profession should test its virtues in practice. We publish an interesting article on the subject, in this number, by Dr. Green, of Macon, Ga.

